# Understanding unemployment: a sociological analysis of systemic challenges and social consequences

**DOI:** 10.3389/fsoc.2025.1674918

**Published:** 2026-01-08

**Authors:** Fernando Fredi Rea García, Sheila Janet Rangel Gómez, Rommel Sebastián Coba Torres, José Luis Domínguez Caiza

**Affiliations:** Faculty of Jurisprudence, Social Sciences, and Politics, State University of Bolívar, Guaranda, Ecuador

**Keywords:** unemployment, symbolic capital, anomie, risk society, social cohesion, social stratification

## Abstract

This article conducts an exhaustive examination of the phenomenon of unemployment from an extensive sociological perspective, emphasizing its structural, cyclical, and multidimensional characteristics. It affirms that unemployment does not solely affect marginalized groups but permeates all social strata and has significant repercussions on social cohesion, mental health, and overall well-being. The ramifications of unemployment go beyond the economic realm and influence vital institutions such as family, education, commerce, and even national security, thereby reinforcing its role as a destabilizing element of social fabric. Methodologically, a non-experimental, interconnected quantitative approach based on the analysis of secondary data on national employment, specifically pertaining to February 2025, is adopted. The need for an integrated approach to understanding this phenomenon is emphasized, given its intricate nature that cannot be elucidated from a singular perspective. In this context, a series of sociological theories are incorporated to broaden the analysis. From Karl Marx’s point of view, unemployment has a functional purpose within the capitalist framework, acting as a social regulation mechanism. Emile Durkheim, on the other hand, introduces the concept of anomie to explain the social disconnection caused by scarce opportunities. Pierre Bourdieu discusses the notion of capital (economic, social, and symbolic) to elucidate how structural inequalities limit access to employment. Ulrich Beck and Zygmunt Bauman, using contemporary frameworks, highlight job precariousness and unpredictability as characteristics of the risk society and liquid modernity. The discussion approaches unemployment as a multifactorial causal factor in society, linked to inequality, insecurity, and anomie. Ultimately, the article presents a comparative analysis of various theoretical propositions across different temporal contexts, constructing a solid framework that facilitates understanding unemployment as a structural phenomenon deeply rooted in the dynamics of contemporary society.

## 1 Introduction

From a contemporary sociological perspective, unemployment constitutes a structural phenomenon that affects social groups in heterogeneous ways while revealing profound inequalities associated with economic, educational, symbolic, and territorial capital. In the Ecuadorian case, official data from the National Employment, Unemployment, and Underemployment Survey (ENEMDU, February 2025) indicate that adequate and full employment rates continue to exhibit significant gaps between urban and rural areas, between men and women, and among individuals with different levels of schooling. Nevertheless, despite the relevance of these figures, national and regional literature presents a persistent analytical gap: most studies have focused on macroeconomic dimensions or descriptive approaches without integrating a sociological analysis that adequately articulates social structure, forms of capital, and the processes of normative disengagement linked to the labor market. In this context, the present study aims to provide recent empirical evidence through a quantitative analysis of ENEMDU 2025, supported by a sociological theoretical framework that interprets unemployment patterns based on the contributions of Pierre Bourdieu and Émile Durkheim.

In the Ecuadorian context, data from the National Employment, Unemployment, and Underemployment Survey (ENEMDU, February 2025) show that unemployment rates exhibit substantial differences between urban and rural areas, as well as between men and women. Likewise, individuals with higher levels of education are not exempt from labor precariousness, revealing a structural pattern that transcends the traditional economic reading of the labor market. It is also important to acknowledge that unemployment reproduces inequalities across different social groups including dimensions such as race, ethnicity, gender, and social class as highlighted by recent studies on stratification and inequality (Darity and Ruiz, 2024). However, despite the availability of national indicators, Ecuadorian and regional literature continues to focus primarily on macroeconomic analyses or general descriptive approaches, without incorporating a holistic examination that considers the multiple factors influencing the phenomenon (Chetty et al., 2017), nor a sociological analysis that articulates the unequal distribution of economic, social, and symbolic capital or the processes of normative disintegration associated with prolonged unemployment (Buheji, 2019). This conceptual and methodological gap reinforces the need to develop empirical studies that integrate robust theoretical frameworks with detailed analyses of the phenomenon, following classical and contemporary contributions that have significantly advanced the understanding of unemployment (Lindbeck, 1991).

In this regard, the novelty of the present study lies in offering a sociological interpretation of unemployment in Ecuador through the integration of empirical evidence from ENEMDU 2025 with the theoretical contributions of Pierre Bourdieu and Émile Durkheim. This approach aligns with the need to situate the phenomenon within the various paradigms that have systematically addressed it, incorporating a synthesis of social perspectives and broad theoretical evaluations developed by significant and widely recognized sociological authors (Sasaki et al., 2014). This proposal makes it possible to simultaneously analyze the structural inequalities linked to different forms of capital and the anomic processes associated with detachment from the labor market, providing a broad and detailed framework for understanding a complex social phenomenon (Didmanidze et al., 2023). Accordingly, the objectives of the study are: (a) to identify the sociodemographic and territorial factors associated with unemployment in the country; (b) to interpret the observed patterns through a sociological framework capable of explaining both structural inequalities and the subjective manifestations of the phenomenon; and (c) to provide recent empirical evidence that contributes to understanding the complexity of unemployment as an evolving social issue beyond its economic dimensions.

The relevance of unemployment in an individual’s economic sphere is undeniable; however, its impact significantly extends to the interactions of these people with the surrounding society (Lindbeck, 1991). This phenomenon is not limited solely to income or expenses, even if the affected person and their family have access to unemployment benefits (Sajjadi et al., n.d.). It also involves significant changes in their employment status, such as transitioning from the desirable role of “contributing citizen” to the less favored condition of “public burden,” accompanied by various moral and stigmatizing consequences (Schöb, 2016). Beyond individual preferences, human beings possess a social dimension, as their behaviors have consequences on the well-being of others within the social framework, which, in turn, influences their own well-being (Anayet Hossain and Korban Ali, 2014). An increase in commerce, for instance, can benefit some groups but harm others; similarly, technological advances can give rise to an apparent “spontaneous generation” of unemployed individuals without direct human intervention (Shi, 2024). Addressing unemployment involves, on one hand, recognizing the reality of the employment situation and, on the other, more or less accurately identifying its fundamental or immediate causes, as well as the economic laws linking these events (Unemployment, 2024).

To sociologically interpret the phenomenon of unemployment in Ecuador, this study adopts a theoretical framework centered on two core approaches: Pierre Bourdieu’s theory of capital and Émile Durkheim’s theory of anomie. This conceptual delimitation responds to the need to avoid excessively broad theoretical panoramas and to ensure coherence between the analytical foundation, the empirical variables employed, and the resulting findings. From Bourdieu’s perspective, the study examines the structural inequalities associated with the distribution of economic, social, cultural, and symbolic capital, a notion consistent with sociological approaches that understand unemployment as a manifestation of inequality and social stratification (Benach and Muntaner, 2024). This perspective also aligns with structural analyses that emphasize how economic development patterns condition the labor market and produce differentiated forms of exclusion (Erickson, 2020; Şiklar and Bilgin, 2020; Litvina, 2022). Concurrently, the Durkheimian perspective on anomie provides a framework for interpreting the subjective and collective processes of normative disintegration, loss of expectations, and weakening of social integration that emerge in situations of prolonged unemployment, especially in contexts where structural transformations reshape the configuration of unemployed populations (Butkaliuk et al., 2022). The combination of both approaches offers a rigorous sociological lens that aligns coherently with the empirical nature of the data analyzed.

From an analytical standpoint, Marx’s work is regarded as one of the foundational bases in sociological theories that address employment and unemployment dynamics (Romanova, 2023). His approach is characterized by a predominantly clear economic conception, where the interaction between capital and labor depends on the availability of an unemployed workforce willing to accept reduced wages. This relationship allows capitalist employers to obtain surplus value. Consequently, wage precariousness and labor inactivity limit access for the unemployed and their families to essential goods, negatively affecting their overall well-being and impacting areas such as health, education, and participation in recreational and sports activities. These conditions give rise to social problems reflected in current definitions of “unemployment” and “social exclusion,” highlighting the severity of the situation. For Marx, individuals are “subjected” to the logic of work and are defined in economic terms as workers, which implies a conception of their essence as a form of degradation. It is through this labor relation that their subjugation manifests.

Human capital can be understood as the complete set of competencies, both cognitive and physical, that an individual possesses, which are essential for their capacity to generate wealth and contribute to the economy (Auerbach and Green, 2024). This conceptualization encompasses not only the knowledge, skills, and talents that people develop but also their potential to turn these resources into effective attitudes and productive behaviors (Anis and Ari, 2024). These manifestations can materialize through various work activities, whether within the framework of paid employment in a company or through self-employment initiatives like entrepreneurship (Gimranova et al., 2024). Recognizing that unemployment generates adverse effects both socially and individually, it is imperative that each person strives to avoid falling into this disadvantage within the labor market.

This can be achieved, for example, through continuous updating and the development of specific skills demanded by a perpetually transforming labor market. From an individual perspective, this approach offers a more optimistic outlook on the unemployment phenomenon, as it encourages those unemployed to consider that, through educational efforts and acquiring additional qualifications, they can successfully reintegrate into the labor market (de Albuquerque Junior et al., 2024). Moreover, when examining various employment situations, it could be argued that unemployment in underdeveloped economies or those on the path to semi-industrialization might not be as worrisome, since inactivity could be seen as a less favorable option compared to the benefits of temporary unemployment.

Each period analyzed in relation to human capital constitutes a distinctly microeconomic approach, seeking to unravel the dynamics of unemployment (Rothomi and Rafid, 2023). More precisely, this perspective focuses on levels of employment and unemployment based on individual behaviors and decisions (Mayilyan and Yedigaryan, 2022). However, a significant limitation of this theory is its inadequate articulation of the fact that the level of employment, as observed and analyzed by various social agents, is ultimately a collective phenomenon resulting from a set of causes impacting the entire economy (Musthafa et al., 2024). Unlike traditional macroeconomic models, which suggest the existence of autonomous and spontaneous unemployment, heterodox economic currents advocate going beyond this reduction to the spontaneous nature of unemployment, considering other factors that influence its emergence.

The study interprets unemployment in Ecuador through a theoretical framework centered on Pierre Bourdieu and Émile Durkheim, ensuring coherence between theory and empirical evidence. From Bourdieu’s perspective, unemployment is explained by the unequal distribution of economic, cultural, social, and symbolic capital, whose effects are reflected in variables such as housing, education, gender, and area of residence dimensions that express differentiated structural positions conditioning access to employment. Likewise, the statistical results such as the higher probability of unemployment among individuals living in precarious housing demonstrate these accumulated inequalities. From Durkheim’s viewpoint, anomie helps explain the subjective dimensions of unemployment, particularly demotivation, institutional distrust, and the perception of lacking opportunities, which emerge when social norms lose their regulatory capacity. The articulation of both approaches makes it possible to simultaneously interpret the structural inequalities and subjective effects associated with unemployment, providing a solid theoretical basis fully aligned with the empirical analysis conducted.

## 2 Materials and methods

The present study employs a quantitative, non-experimental, and cross-sectional design, based on secondary data from the National Employment, Unemployment, and Underemployment Survey (ENEMDU, February 2025). This survey is produced by the National Institute of Statistics and Censuses (2025) (INEC) of Ecuador following methodological standards established by the International Labour Organization (ILO), which ensures the international comparability of labor indicators. ENEMDU uses probabilistic, stratified, and multistage sampling, with field supervision procedures, consistency checks, and controls for non-sampling errors; therefore, its validity is institutional, and its reliability is supported by the technical standardization applied by INEC throughout all stages of data collection.

The selected variables were operationalized in accordance with INEC’s methodological guidelines. Structural variables educational level, type of housing, area of residence, and gender were incorporated into the analysis as empirical indicators of the forms of economic, cultural, social, and symbolic capital described by Pierre Bourdieu, allowing for an interpretation of inequalities in labor market insertion. In turn, the “reasons for not seeking employment” were included as empirical approximations of Durkheimian anomie, as they reflect perceptions of hopelessness, institutional distrust, or lack of opportunities associated with weakened social integration.

To assess associations between categorical variables, the Chi-square test was applied an appropriate technique for determining independence or dependence between categories without requiring parametric assumptions. Subsequently, a binary logistic regression model was performed to estimate the probability of being unemployed as a function of predictors selected for their sociological relevance and their recurrent presence in the literature on labor inequalities: gender, educational level, area of residence, and housing type. The resulting coefficients (odds ratios) make it possible to interpret the relative weight of each structural factor in unemployment, linking the findings to the sociological approach of the study.

The interpretation of results was conducted by integrating empirical evidence and sociological theory, following the analytical logic proposed by Bourdieu and Durkheim, which ensures coherence between the theoretical framework, statistical analysis, and discussion of findings.

## 3 Results

The academic data focus on the correlation between housing types and the occupational conditions of the Ecuadorian population, distinguishing between urban and rural contexts (National Institute of Statistics and Censuses, 2022). The goal is to comprehensively understand the residential environment of individuals (National Institute of Statistics and Censuses, 2023). To this end, a frequency table generated from data supplied by the National Employment, Unemployment, and Underemployment Survey (ENEMDU) of February 2025 is employed. The research facilitates the observation of how habitat characteristics, regardless of whether they are located in urban or rural areas, may be related to individuals’ labor circumstances, thus providing important empirical evidence on the distribution of economic and social capital across various sectors of the country.

Table 1 shows that 58.1% of the participants reside in houses or villas, representing the most significant data point in the group, while 31.2% live in apartments. This distribution not only indicates a preference in housing selection but also reflects a broader structural dynamic. From the theoretical framework proposed by Pierre Bourdieu, the category of housing can be interpreted as a manifestation of the economic and symbolic capital possessed by individuals, which indicates their position within the social hierarchy (Bozlu, 2020). Living in houses or villas can be viewed as an indicator of increased economic stability and status aspirations, which in turn reinforces pre-existing social inequalities (Schofield, 2021).

**Table 1:** Housing type.

Frequency	Percentage	Valid percentage	Cumulative percentage
Valid	House or villa	5,094	58.1
	Apartment	2,733	31.2
	Room in a boarding house	240	2.7
	Shack	221	2.5
	Hut or makeshift shelter	472	5.4
	Shack	6	0.1
Total	8,766	100.0	100.0

Original work based on data obtained from the National Employment and Underemployment Survey (ENEMDU), February 2025.

An analysis of correlation was conducted to investigate the association between gender and unemployment status in February 2025. Empirical data indicate that 51.50% of men with advanced educational qualifications are unemployed, while for women, this percentage increases to 48.50%. Using Pierre Bourdieu’s sociological framework, this phenomenon can be explained through the unequal distribution of social and symbolic capital (Estides Delgado, 2021). Despite possessing educational capital, many individuals are unable to penetrate the labor market due to social structures that perpetuate gender and class disparities in access to opportunities (Kleanthous, 2022).

Regarding the relationship between education level and unemployment, data from February 2025 show that 42.80% of unemployed individuals have completed secondary education or baccalaureate studies. However, a very close figure 42.37% corresponds to people with higher education. This trend reflects a concerning pattern, in line with Ulrich Beck’s modern sociological theory and his concept of the “risk society.” This situation demonstrates that even those with educational credentials are not exempt from job precariousness. Education no longer guarantees stability, as current economic structures generate uncertainty and instability across all social levels.

A statistical correlation analysis was conducted to elucidate the factors contributing to the phenomenon of some individuals abstaining from actively seeking employment. The most significant results reveal that 45.33% of respondents do not search for work actively because they believe they cannot secure a position, while 33.33% state that they see no viable employment opportunities. Based on Émile Durkheim’s theory of anomie, this scenario can be interpreted as a manifestation of the disintegration of the connection between social aspirations and the legitimate means to achieve them. This situation generates feelings of frustration, resignation, and demotivation toward a system that does not offer substantial guarantees for integration into the labor market.

### 3.1 Logistic regression analysis: predictors of unemployment

To identify the factors that significantly influence the likelihood of being unemployed, a binary logistic regression analysis was conducted. The results are presented in Table 2.

**Table 2:** Logistic regression model for unemployment predictors.

Predictor variable	*B*	Standard error	Wald	df	Sig.	Exp (*B*)	95% confidence interval for Exp (B)
Sex (ref: male)	0.426	0.112	14.442	1	0.000^**^	1.531	1.230–1.907
Education level		18.726	3	0.001^**^			
Primary (ref)							
Secondary	0.175	0.156	1.257	1	0.262	1.191	0.877–1.618
Higher	0.583	0.164	12.635	1	0.000^**^	1.791	1.299–2.471
Postgraduate	0.396	0.237	2.785	1	0.095	1.486	0.934–2.364
Area (ref: urban)	−0.328	0.129	6.464	1	0.011^*^	0.720	0.559–0.927
Housing Type		12.841	5	0.025^*^			
House/Villa (ref)							
Apartment	0.053	0.119	0.198	1	0.656	1.054	0.835–1.331
Room in boarding house	0.397	0.198	4.027	1	0.045^*^	1.487	1.009–2.192
Shack (mediagua)	0.456	0.201	5.146	1	0.023^*^	1.578	1.064–2.340
Shack or makeshift shelter	0.517	0.148	12.195	1	0.000^**^	1.677	1.254–2.243
Hut	0.624	0.489	1.627	1	0.202	1.866	0.715–4.872
Constant	−2.369	0.177	179.192	1	0.000	0.094	–

Original work based on data obtained from the National Employment and Underemployment Survey (ENEMDU), February 2025. ^*^p < 0.05, ^**^p < 0.01 *R*^2^ de Nagelkerke = 0.084; Hosmer–Lemeshow: *χ*^2^ = 9.237, gl = 8, *p* = 0.323.

The logistic regression model was statistically significant (*χ*^2^ = 167.532, df = 10, *p* < 0.001), explaining approximately 8.4% of the variance in unemployment status (Nagelkerke *R*^2^ = 0.084). The Hosmer–Lemeshow goodness-of-fit test indicates an adequate model fit (*p* > 0.05). Results reveal that, controlling for other variables, women have a 53.1% higher likelihood of being unemployed compared to men (OR = 1.531, *p* < 0.001). Concerning education level, contrary to conventional expectations, individuals with higher education show a 79.1% greater probability of unemployment than those with only primary education (OR = 1.791, *p* < 0.001). This finding supports Beck’s concept of labor precarity in the risk society, where even higher education does not guarantee employment stability. Regarding geographic location, residing in rural areas is associated with a 28% decrease in the probability of unemployment compared to urban areas (OR = 0.720, *p* = 0.011), suggesting different labor dynamics across contexts. Finally, regarding housing type, living in precarious units such as shacks or makeshift homes significantly increases the likelihood of unemployment (OR = 1.677, *p* < 0.001) compared to those living in houses or villas, reinforcing Bourdieu’s theory about the interconnectedness of different forms of capital.

### 3.2 Analysis of unemployment duration and differential effects

A key aspect to understanding the depth of the impact of unemployment is analyzing its duration. Table 3 presents the distribution of the unemployed population according to the length of their job search.

**Table 3:** Distribution of the unemployed population by duration of unemployment.

Duration of unemployment	Frequency	Percentage	Cumulative percentage
Less than 1 month	127	12.4%	12.4%
1–3 months	284	27.8%	40.2%
4–6 months	249	24.4%	64.6%
7–12 months	186	18.2%	82.8%
More than 12 months	176	17.2%	100.0%
Total	1,022	100.0%	

Original work based on data obtained from the National Employment and Underemployment Survey (ENEMDU), February 2025.

**Figure 1:**
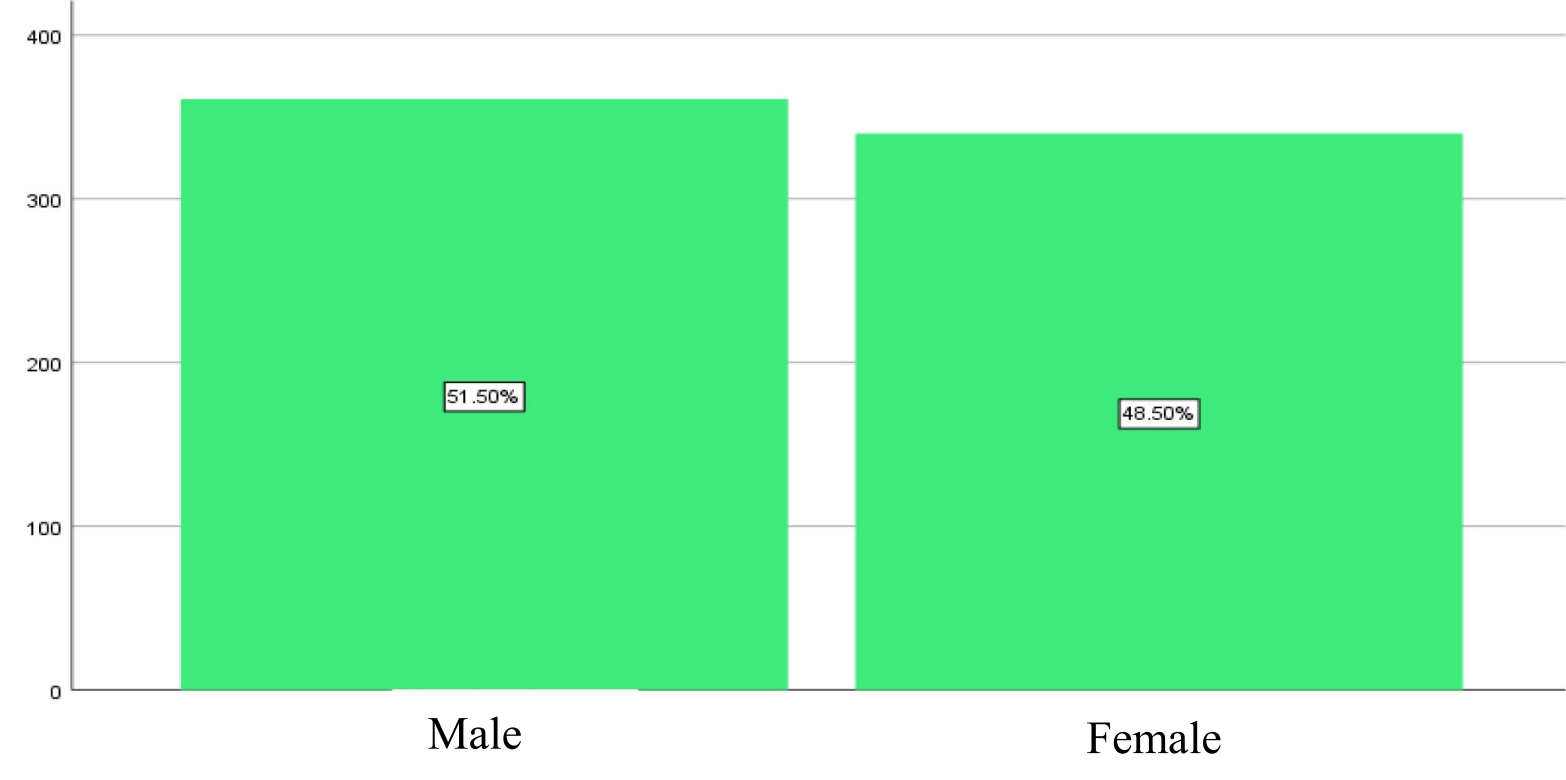
Unemployment by gender in Ecuador. Original work based on data obtained from the National Employment and Underemployment Survey (ENEMDU), February 2025.

The data reveal that over one-third (35.4%) of the unemployed population has remained in this condition for more than 6 months, a period considered the threshold for long-term unemployment according to international standards. Additionally, 17.2% have experienced chronic unemployment exceeding 1 year, a situation that substantially increases the risk of social anomia, as Durkheim proposed, due to the prolonged disconnection from the normative structures of work. When analyzing unemployment duration by education level (Figure 1), an unexpected pattern emerges: individuals with higher education experience longer periods of unemployment (average = 7.8 months) than those with primary education (average = 5.2 months). This finding contradicts the conventional notion of human capital and reinforces Beck’s theory of increasing precariousness in the risk society.

### 3.3 Impact of psychosocial aspects of unemployment: qualitative narrative analysis

**Figure 2:**
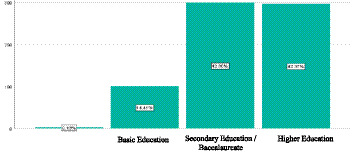
Educational level. Original work based on data obtained from the National Employment and Underemployment Survey (ENEMDU), February 2025.

**Figure 3:**
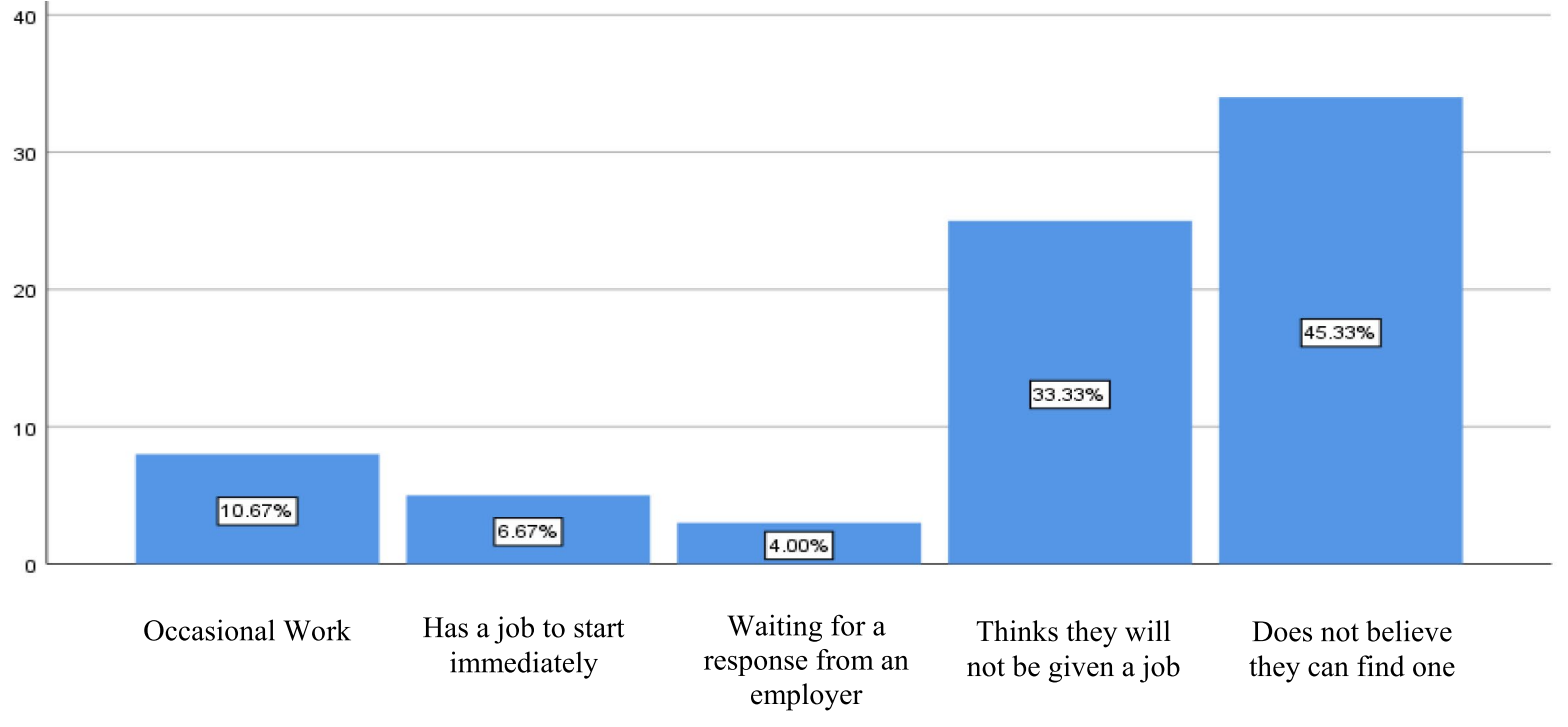
Reasons why people do not search for work in Ecuador. Original work based on data obtained from the National Employment and Underemployment Survey (ENEMDU), February 2025.

To complement the quantitative analysis, qualitative responses regarding reasons for not seeking employment were examined. This analysis enabled the identification of discursive patterns associated with different manifestations of social anomy, in line with Durkheim’s conceptualization. Table 4 presents the main patterns identified through content analysis of the open-ended responses (Figures 2, 3).

**Table 4:** Discursive patterns in narratives about not searching for employment.

Discursive pattern	Description	Frequency	Percentage	Representative example
Learned helplessness	Expressions of resignation after repeated failures	127	32.5%	“I’ve tried many times and they never call back.”
Institutional distrust	Questioning the legitimacy of the labor system	89	22.8%	“Good jobs are only for those with contacts.”
Social isolation	Loss of support networks for job searching	76	19.5%	“I do not know anyone who could recommend me.”
Value reorientation	Rejection of work as the central axis of identity	62	15.9%	“I prefer to do other things that make me feel more fulfilled.”
Normalized precariousness	Acceptance of informal conditions as inevitable	36	9.2%	“Why bother searching if they only offer low-paid jobs?”

Original work based on data obtained from the National Employment and Underemployment Survey (ENEMDU), February 2025.

These discursive patterns reflect different manifestations of Durkheim’s concept of anomie, ranging from the rupture of social integration (social isolation) to the erosion of normative regulation (institutional distrust). When analyzing these patterns by education level, it was found that learned helplessness is most prevalent among individuals with higher education (41.3%), while normalized precariousness is more common among those with primary education (24.7%). This differential distribution suggests that the subjective experience of unemployment is mediated by social expectations linked to the accumulated cultural capital.

## 4 Discussion

The empirical results reveal structural patterns that enable an integrated sociological understanding of unemployment in Ecuador. First, the logistic regression model shows that the highest risk of unemployment is concentrated in three dimensions: individuals with higher education (OR = 1.791, *p* < 0.001), those living in precarious housing such as shacks or makeshift dwellings (OR = 1.677, *p* < 0.001), and women (OR = 1.531, *p* < 0.001). These three variables represent inequalities in the accumulation of economic, cultural, and symbolic capital, consistent with Bourdieu’s theoretical framework and with contemporary analyses demonstrating that structural labor dynamics reinforce unequal distributions of opportunity (Stavila and Vaidean, 2023). Thus, holding educational credentials does not necessarily translate into labor market advantages when individuals lack effective social networks or face symbolic delegitimization in the labor market.

Similarly, the analysis of the reasons for not seeking employment reveals that 45.33% of individuals refrain from job searching because they believe they will not find work, while 33.33% perceive a complete absence of opportunities. These responses constitute empirical expressions of anomie, understood in Durkheimian terms as the weakening of normative structures that guide expectations and actions. This interpretation aligns with classical sociological perspectives arguing that work, meaning, and social integration are deeply interdependent elements within capitalist societies (Knight, 1979). Feelings of resignation, institutional distrust, and demotivation reflect processes of social disintegration associated with interrupted or precarious labor trajectories.

The comparison between urban and rural areas indicates that living in rural zones functions as a protective factor against unemployment (OR = 0.720, *p* = 0.011), suggesting differentiated productive dynamics and potentially denser forms of social capital in rural territories. This finding further reinforces the idea that labor transitions are conditioned by social and territorial structures rather than by isolated individual decisions.

### 4.1 Housing as an indicator of economic and symbolic capital

The initial pertinent observation pertains to the distribution of housing types among participants: 58.1% substantially reside in houses or villas, while 31.2% live in apartments. Based on Pierre Bourdieu’s theoretical framework, this housing preference transcends mere quantitative data (Rogošić and Baranović, 2016). Within his conceptualization of habitus and the various forms of capital, namely economic, cultural, social, and symbolic, housing functions as a representation of symbolic capital and serves as a tangible expression of an individual’s position within the social hierarchy (de Souza and Fenili, 2016). In this sense, individuals residing in houses or villas tend to possess greater economic capital or aspire to a more entrenched social status, thereby perpetuating a cycle of social inequalities. This differentiation in housing further facilitates an indirect correlation with employment prospects: people with higher levels of economic capital often possess superior social networks (social capital) and higher educational qualifications (cultural capital), which improve their accessibility to the labor market (Rico Rios, 2020).

Consequently, analyzing housing emerges as a fundamental aspect for exploring structural disparities related to access to employment and job security. The pertinent data from the analysis of housing typology shows that 58.1% of participants live in houses or villas, while 31.2% reside in apartments. Relying on Pierre Bourdieu’s theoretical framework, this preference for housing goes beyond simple quantitative observation. In his concepts of habitus and capital (encompassing economic, cultural, social, and symbolic dimensions), housing constitutes a form of symbolic capital, an observable expression of a person’s position within the social hierarchy (Fatmawati, 2020). Therefore, individuals living in houses or villas are predisposed to possess greater economic capital or to aspire toward a more deeply rooted social status, which contributes to the reproduction of social inequalities (Jenkins, 2024).

### 4.2 Gender inequality and unemployment: a matter of symbolic capital

Regarding the correlation between gender and unemployment, empirical data reveal that 51.5% of men with advanced educational qualifications are unemployed, compared to 48.5% of women at the same educational level. Despite the relatively marginal difference, this statistic prompts a critical reflection on the effectiveness of educational capital in guaranteeing employment. Once again, Bourdieu’s theoretical perspective stands out, as it warns that educational capital does not invariably translate into tangible advantages unless complemented by other forms of capital, such as social or symbolic capital (Oliveira and da Silva, 2022). In this context, women may suffer from various forms of structural discrimination that hinder their employability, regardless of their high educational levels. The persistence of gender stereotypes, labor market segmentation, and the burden of family responsibilities negatively affect their symbolic capital and restrict access to prestigious or stable jobs (Jain, 2020). The theory of social reproduction further clarifies that these inequalities are not incidental but arise from persistent logics inherent to the capitalist system, which perpetuate exclusion through institutionalized mechanisms.

### 4.3 Education and job insecurity: the dissolution of the meritocratic contract

The data from the study reveal a concerning proximity between the unemployment rates of individuals with secondary education (42.8%) and those with higher educational degrees (42.37%). This conclusion fundamentally challenges the legitimacy of the meritocratic principle, which claims that diligent academic participation and credential acquisition guarantee social mobility and economic security (Babacan, 2024). This assertion has been extensively debated among contemporary sociologists, including Ulrich Beck, who, through his framework of a “risk society,” argues that the social structures once providing stability have disintegrated (Gilleard and Higgs, 2024). In risk-laden societies, job insecurity and instability affect all social classes, including those with high academic qualifications. Education no longer functions as a safeguard against precarity; instead, in some cases, it can lead to disillusionment when it fails to fulfill aspirations of social ascent. Zygmunt Bauman also explored this phenomenon through his concept of liquid modernity, where institutions lose power and uncertainty emerges as a pervasive reality (Akbaş, 2024). Educated youth now face an adaptable, unpredictable, and competitive labor market, in which academic credentials no longer guarantee employment, let alone a stable position.

### 4.4 Anomia, hopelessness, and disconnection from the labor market

Another relevant observation is that 45.33% of participants who do not actively seek employment attribute their inaction to the belief that they cannot find a job, while 33.33% perceive a lack of viable opportunities. This phenomenon can be examined through Émile Durkheim’s theory of anomie. According to this foundational sociologist, anomie arises when the social norms that regulate behavior diminish in strength, leaving individuals without a definitive moral or institutional framework. In the context of unemployment, this erosion of reference points manifests as a detachment of the population from the labor market, accompanied by feelings of frustration, resignation, and demotivation (Chapsal, 2022).

Furthermore, Durkheim proposed that anomie could lead to “anomic suicide,” a form of social despair where the individual retreats from the social fabric (Xinyu, 2023). While this scenario does not reach such extreme levels, milder manifestations of disconnection can be observed, indicating a lack of integration into the economic system. This analysis can be further clarified through the concept of structural unemployment, extensively articulated by contemporary theorists, who warn that many economies systematically produce a surplus of unemployed individuals who cannot find their place, not due to lack of capacity, but as a direct consequence of the structural configuration of the market itself (Faizi and Nayebi, 2023).

### 4.5 Unemployment as a manifestation of structural inequality

The statistical framework surrounding unemployment not only illuminates the data but also clarifies the fundamental structures that explain and perpetuate this phenomenon from the classical theoretical perspective of Karl Marx. According to Marx, unemployment can be understood as a mechanism employed by the capitalist system to regulate the workforce and keep wage levels at a minimum threshold (McNeill, 2024). The concept of an “industrial reserve army” serves a functional role for capital, ensuring a continuous supply of workers willing to accept precarious working conditions (Juwaini et al., 2024). This reasoning is especially pronounced in situations where even individuals with specialized skills are unemployed, thereby undermining their ability to negotiate effectively.

Additionally, the data reflecting gender, class, and geographic disparities align with Marxist critiques of the dominant economic structure (Du Bois, 2021). The concentration of capital among a privileged few, the commodification of labor, and the normalization of social inequalities are systemic processes that clarify why unemployment is not merely a random fact but a structural reality (Easton, 2023). Furthermore, Weberian critique emphasizes the influence of cultural values, religious beliefs, and hegemonic ideologies in shaping employment opportunities and individual life trajectories, thus perpetuating inherited advantages (de Faria, 2021).

## 5 Conclusion

The study demonstrates that unemployment in Ecuador responds to structural inequalities linked to the unequal distribution of economic, cultural, and symbolic capital, rather than to individual deficiencies or lack of training. Individuals with higher education, women, and those living in precarious housing exhibit higher probabilities of unemployment, indicating that the accumulation of cultural capital does not guarantee labor mobility when it is not accompanied by social and symbolic capital. Likewise, the subjective reasons for not seeking employment reveal clear manifestations of anomie, expressed in perceptions of distrust, frustration, and the absence of legitimate opportunities, indicating a weakening of the mechanisms of social integration. This suggests that unemployment generates not only economic effects but also normative and symbolic ones. Taken together, the findings provide a robust sociological framework for understanding unemployment as a multidimensional phenomenon. The articulation between statistical analysis and sociological theory constitutes the principal contribution of this study, offering updated empirical evidence consistent with the dynamics of the Ecuadorian labor market. These results may guide future public policies aimed at reducing structural inequalities and strengthening mechanisms of labor and social integration.
